# Epidemiology of functional shoulder instability: an online survey

**DOI:** 10.1186/s12891-019-2563-7

**Published:** 2019-06-11

**Authors:** Victor Danzinger, Eva Schulz, Philipp Moroder

**Affiliations:** 1 Department of Shoulder and Elbow Surgery, Center for Musculoskeletal Surgery, Charité - Universitaetsmedizin Berlin, Campus Virchow Klinikum, Augustenburger Platz 1, 13353 Berlin, Germany; 2 Department of Traumatology and Sports Injuries, Kardinal Schwarzenberg Klinikum, Schwarzach im Pongau, Austria; 3 Teaching Hospital of the Paracelsus Medical University, Salzburg, Austria

**Keywords:** Controllable functional shoulder instability, Voluntary dislocation, Positional instability, Cross sectional study, Prevalence

## Abstract

**Background:**

Functional shoulder instability (FSI) is defined as glenohumeral instability that is not caused by structural defects but rather by abnormal muscle activation patterns. Patients with FSI are able to dislocate their shoulder at will, either by motion (positional FSI) or even without moving the arm (non-positional FSI). In contrast to structural shoulder instability, little is known about the epidemiology of FSI. The aim of the following study was to further analyse this rare pathology and approximate the prevalence of FSI.

**Methods:**

A self-evaluated and anonymous online survey among 5866 medical students was conducted using the students email list of two german-speaking medical universities (Study Center 1 and Study Center 2). Possibly affected siblings were used as a supplementary group (Siblings Cohort). General sociodemographic data, dislocation mechanism, potential causes, age at the tie of developing first symptoms, general hyperlaxity, previous interventions, and sporting activity were evaluated and analyzed. The total number of email recipients and responses was used to estimate the minimal and maximal prevalence of FSI.

**Results:**

Five hundred thirteen questionnaires were completed by the students and subsequently analyzed. In total, there were 32 participants with FSI. The minimal prevalence of FSI was found to be 0,5% and the maximal prevalence 2,6%. In most cases (67%) a positional FSI was reported. The majority of the patients reported that first symptoms developed under the age of 16 years (69%) and without any traumatic event (72%). Most of the affected participants had no therapeutic intervention for their FSI (69%) and performed non-overhead (59%) or overhead sports (28%).

**Conclusion:**

Functional shoulder instability (FSI) is more common than expected amongst young adults and seems to develop during childhood mostly without specific reason.

## Strengths and limitations of this study

- First study to specifically analyze the prevalence of functional shoulder instability.
- A large number of medical students at two different universities participated in this study along with a cohort formed by their siblings.
- Only medical students were included as they can be expected to report more specifically about their pathology.
- Due to the self-evaluated study design, there was no objective clinical evaluation of the pathology.
- The observed prevalence among medical students and their siblings might not directly transfer to the general population.

## Background

To date, shoulder instability in general is a well-known pathology in terms of clinical evaluation, diagnostic imaging and recommended treatment. According to the Stanmore classification, shoulder instability can be separated into three groups based on the cause of instability. Traumatic bony or soft tissue defects can be observed in Polar Type I (traumatic-structural) while atraumatic structural insufficiencies are present in Polar Type II (atraumatic-structural). However, there is also a subtype of shoulder instability which is not caused by structural defects but rather by abnormal muscle activation patterns called Polar Type III [1].

Moroder et al. (unpublished data: Moroder et al. 2019, under review JSES) proposed to call this subtype functional shoulder instability (FSI), as opposed to structural shoulder instability. FSI can sometimes willfully be executed (controllable FSI) which results in no or little functional impairment. However, FSI can also unwillfully cause repetitive shoulder subluxations or dislocations resulting in severe functional impairment (non-controllable FSI) [2]. In most cases, the often young patients develop their symptoms without any trauma [1–3].

While the epidemiology of structural shoulder instability has been extensively studied, very little is known about FSI [4–9]. In 1982, Hovelius interviewed 2092 people using a multistage selection process and found 35 (1.7%) participants with shoulder joint dislocation in their medical history. Voluntary shoulder instability has been described in only 1 bilateral case (0.05%), which can be interpreted as the first epidemiological description of functional shoulder instability [10]. More recently, Malone et al. identified an abnormal muscle patterning in 46% of patients suffering from recurrent shoulder instability indicating a possible higher prevalence of FSI than expected [11].

The aim of our study was to analyze the prevalence of FSI in a young population as pathology-specific epidemiological studies are currently lacking.

## Methods

To assess the prevalence of FSI, a web-based survey among students was conducted. To minimize the limitation of a self-evaluation study, we specifically included only medical students as we assumed they would have a better knowledge and understanding for the pathology itself. The survey was distributed using the students email list of two german-speaking medical universities. The first study center (Study Center 1) was a large medical university based in Germany with around 600 new students per year. The second study center (Study Center 2) was a smaller medical university with approximately 125 new students per year based in Germany and Austria. Overall, the online survey was sent to 5300 medical students of the Study Center 1 and 566 medical students of the Study Center 2 resulting in a total number of 5866 potential participants. Further questions about possibly affected siblings of all interviewed participants led to an additional group of 723 participants (Siblings Cohort).

The total number of possible participants (email recipients) and the total number of interviewed participants (responses) was used to calculate the minimal and maximal prevalence of FSI per study site. The survey period lasted 3 weeks including three consecutive reminders at all study centers.

### Online survey

The anonymous and specifically developed online questionnaire included general sociodemographic data and 16 multiple-choice questions for each participant. The term “Functional shoulder instability” was applied if a participant had the ability to willfully dislocate his shoulder during shoulder movement (positional FSI) or at rest in neutral position (non-positional FSI). Further assessment included potential causes, age at first occurrence of the instability episodes, general hyperlaxity, sporting activity and possible factors leading to the pathology as well as previous conservative or surgical interventions. Furthermore, the total number of siblings and the number of siblings affected by FSI were requested. The web-based study was conducted using SoSci Survey (SoSci Survey GmbH, Munich, Germany). According to the local ethical committee and the local data protection authority no formal approval had to be obtained due to the voluntary nature of the study not involving any patients and anonymous data collection.

## Results

### Demographics

In total, 513 questionnaires were completed. There were 305 (59%) female and 208 (41%) male participants. The mean age was 23 ± 4 years (range: 15–55 years), mean height 174 ± 9 cm (range: 151–200 cm) and mean weight 68 ± 12 kg (range: 44–120 kg). 459 (89%) participants were right handed, 42 (8%) were left handed and 12 (2%) participants stated ambidexterity.

Possibly affected siblings of all participants have been included in the final approximation process. There were 330 subjected siblings at Study Center 1 and 393 siblings at Study Center 2.

Thus, the total number for approximation the maximal prevalence was 1236 with 238 participants (19%) of Study Center 1, 275 (22%) participants of Study Center 2 and 723 (58%) participants regarding the siblings’ cohort.

### Prevalence of FSI

At Study Center 1, 238 of 5300 possible participants (4%) completed the web-based survey. Overall, there were 15 participants with the ability to willfully dislocate their shoulder resulting in a minimal possible prevalence of FSI of 0.3% (15/5300) and a maximal prevalence of 6.3% (15/238).

At Study Center 2, 275 questionnaires were completed of 566 (49%) possible study participants. 9 participants with FSI were detected with consequential minimal prevalence of 1.6% (9/566) and maximal prevalence of 3.3% (9/275).

Further online assessment included possibly affected siblings of all participants. 8 of 723 siblings were reported to have FSI resulting in a maximal prevalence of 1.1% (8/723). The minimal prevalence of the sibling cohort could not be determined due to the study design.

Overall, there were 32 participants with FSI in the total study cohort. Therefore, the minimal prevalence of FSI was 0.5% (32/5866) and the maximal prevalence 2.6% (32/1236) (Table 1).

**Table 1:** Prevalence of FSI for medical students and their related siblings at Study Center 1 and Study Center 2

Prevalence FSI	Study Center 1	Study Center 2	Siblings Cohort	Total
Total Possible Participant	5300	566		5866
Total Interviewed	238	275	723	1236
Total FSI	15	9	8	32
Minimal Prevalence	0.3%	1.6%		0.5%
Maximal Prevalence	6.3%	3.3%	1.1%	2.6%

### Dislocation mechanism

**Fig. 1:**
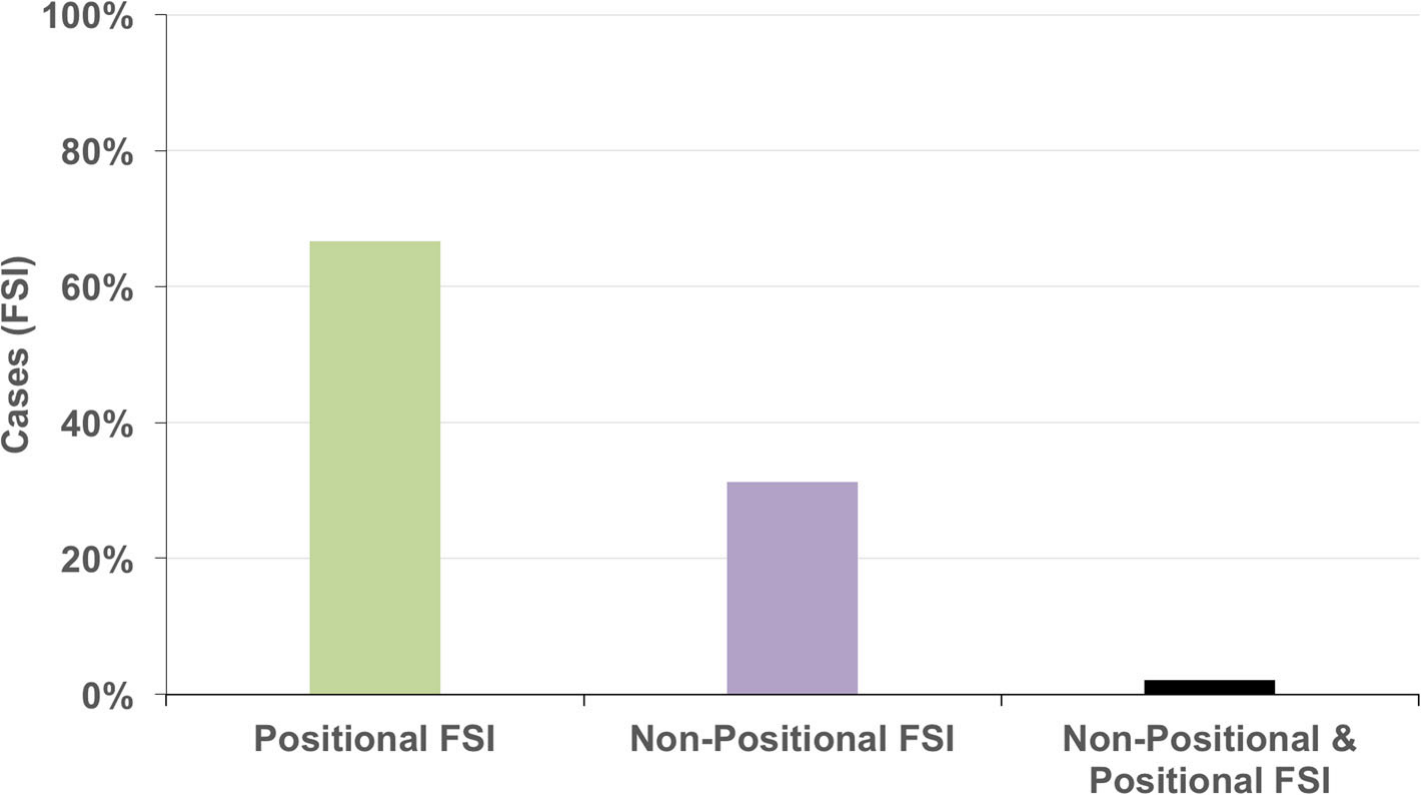
Type of FSI in medical students and their related siblings. Positional FSI occurs during shoulder movement and non-positional FSI at rest in neutral position

Based on the instability mechanism, 16 participants could dislocate one shoulder and 16 were able to dislocate both shoulders resulting in 48 cases of FSI. In the majority of cases (67%) participants were able to willfully dislocate their shoulder during shoulder movement (positional FSI) (Fig. 1).

### Epidemiology and Aetiology

**Fig. 2:**
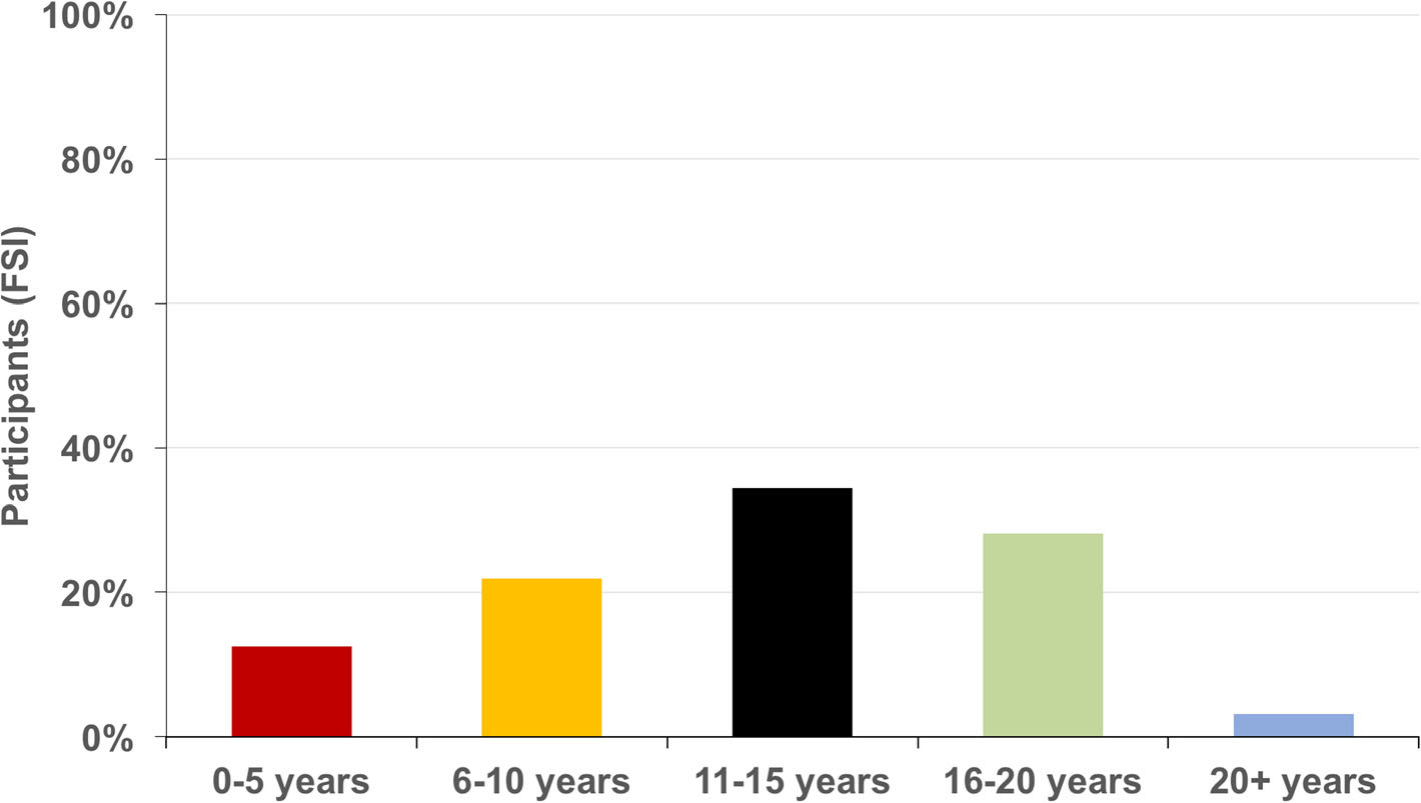
Age of development of the first symptoms of functional shoulder instability (FSI)

Furthermore, 9 (28%) participants stated that first shoulder instability symptoms occurred following a not further specified traumatic event and 23 (72%) stated atraumatic development of FSI. First symptoms developed during childhood (0–15 years) in 22 participants (69%) (Fig. 2). Furthermore, 13 (41%) study participants reported general hyperlaxity.

### Previous treatment

9 (28%) participants affected with FSI have been under prior medical care. 5 (16%) participants stated their shoulder had been reduced in the past and 4 (13%) participants had received immobilization of the affected shoulder. 1 (3%) participant underwent surgery. 9 (28%) participants attempted conservative physiotherapeutic treatment for several weeks (25%) or months (3%) whereas 22 (69%) participants had no prior therapeutic intervention.

### Sports

Assessment of sports activity revealed no sporting activity in 4 (13%), non-overhead sports in 19 (59%) and overhead sports in 9 (28%) participants with FSI. Sports activity level showed no considerable intensity in 3 (9%), less than 1 h per week in 7 (22%), 1–2 h per week in 9 (28%) and over 2 h per week in 13 (41%) participants respectively.

## Discussion

To our knowledge, this study is the first describing the prevalence of functional shoulder instability in a cross sectional study design. Using an online survey is a simple yet comprehensive tool for obtaining a valuable study cohort.

Our main results compare three different study samples and estimate the prevalence of FSI to be between 0.5 and 2.6% for the described population (Table 1). An even larger number of affected participants with FSI is debatable, as subluxation and instant reduction of the shoulder joint is sometimes not noticed as shoulder instability by the affected persons.

### Comparison with existing literature

Regarding the dislocation mechanism of the subjected cases 67% stated positional FSI and 31% reported non-positional FSI including 2% of concurrent positional and non-positional FSI. Equally to these results, Moroder et al. (unpublished data: Moroder et al. 2019, under review JSES) found a higher number of positional instability cases (78%) than non-positional FSI (22%) in a 1-year prospective study characterizing 36 cases with FSI [2]. Thus, we can confirm a higher prevalence rate of positional FSI compared to non-positional FSI. Moreover, atraumatic development was higher (72%) than minor-traumatic (28%) symptom manifestation similar to previous findings [2].

Overall, almost 70% of affected participants with FSI experienced first symptoms in their childhood (0–15 years) and nearly half of the cohort stated hyperlaxity in concordance with previous studies describing the typically adolescent and hypermobile patient with functional shoulder instability (Fig. 2) [1–3, 12, 13].

Further analysis evaluated prior treatment approaches. Generally, the functional component should not be surgically addressed and surgery should be avoided in patients enduring FSI as it can lead to even worse outcome including severe pain, loss in range of motion and contribute to early degenerative processes [3, 12, 14–16]. Conservative treatment is the therapy of choice and should include patients’ education, muscle strengthening, tactile feedback or electric muscle stimulation as promising alternative intervention [3, 12, 17].

### Limitations

One main limitation of our study was the lack of a clinical evaluation for the verification of the pathology. To ensure a large study cohort and offer anonymous data collection, we specifically aimed for including only medical students. Thus, we assumed to have a general knowledge recognizing subluxations and dislocations of the shoulder also including their related siblings to provide an additional study cohort in the analysis. However, we could not assess the total number of all possible related siblings in the approximation process.

Furthermore, our findings indicate a minimal prevalence of FSI of 0.5%, yet it could be slightly lower as participants might have a false understanding of their symptoms or might overinterpret them. Additionally, the presence of large structural defects could enable students to willfully dislocate their shoulders thus mimicking a FSI. However, such extensive structural defects are very rare and usually can be clearly attributed to a painful macro-trauma. Moreover, functional shoulder instability might also be associated with additional structural deficiencies such as glenoid dysplasia [2]. Therefore, future studies should include CT or MRI scanning in their evaluation process.

Another limitation could be a possible selection bias due to the generally young and healthy study population of medical students. Regarding the prevalence of FSI in the general population, the exact prevalence remains unclear.

## Conclusion

The prevalence of functional shoulder instability (FSI) among medical students including their siblings has a range between 0.5 and 2.6%. 67% of the affected cases reported positional FSI and 31% non-positional FSI with 2% of coexisting positional and non-positional FSI. In over two-thirds of the affected persons first instability episodes occurred under the age of 16 years.

## Abbreviation

FSI: Functional shoulder instability

## References

[1] Lewis A, Kitamura T, Bayley JIL (2004). (ii) The classification of shoulder instability: new light through old windows!. Curr Orthop.

[2] Moroder P, Danzinger V, Maziak N, Plachel F, Pauly S, Scheibel M, et al. Characteristics of Functional Shoulder Instability. J Shoulder Elb Surg. accepted for publication 2019. 10.1016/j.jse.2019.05.025 31378683

[3] Takwale VJ, Calvert P, Rattue H (2000). Involuntary positional instability of the shoulder in adolescents and young adults. Is there any benefit from treatment?. J Bone Joint Surg Br.

[4] Tas M, Canbora MK, Kose O, Egerci OF, Gem M (2013). Demographic and clinical characteristics of traumatic shoulder dislocations in an urban city of Turkey: a retrospective analysis of 208 cases. Acta Orthop Traumatol Turc.

[5] Kroner K, Lind T, Jensen J (1989). The epidemiology of shoulder dislocations. Arch Orthop Trauma Surg.

[6] Shah A, Judge A, Delmestri A, Edwards K, Arden NK, Prieto-Alhambra D (2017). Incidence of shoulder dislocations in the UK, 1995-2015: a population-based cohort study. BMJ Open.

[7] Milgrom C, Mann G, Finestone A (1998). A prevalence study of recurrent shoulder dislocations in young adults. J Shoulder Elb Surg.

[8] Liavaag S, Svenningsen S, Reikeras O, Enger M, Fjalestad T, Pripp AH (2011). The epidemiology of shoulder dislocations in Oslo. Scand J Med Sci Sports.

[9] Zacchilli MA, Owens BD (2010). Epidemiology of shoulder dislocations presenting to emergency departments in the United States. J Bone Joint Surg Am.

[10] Hovelius L. Incidence of shoulder dislocation in Sweden. Clin Orthop Relat Res. 1982;(166):127–31. 7083659

[11] Malone A, Jaggi A, Calvert P, Lambert S, Bayley I (2005). The prevalence of inappropriate muscle sequencing in recurrent shoulder instability. Orthopaedic Proceedings.

[12] Jaggi A, Lambert S (2010). Rehabilitation for shoulder instability. Br J Sports Med.

[13] Rowe CR, Pierce DS, Clark JG (1973). Voluntary dislocation of the shoulder. A preliminary report on a clinical, electromyographic, and psychiatric study of twenty-six patients. J Bone Joint Surg Am.

[14] Hawkins RJ, Koppert G, Johnston G (1984). Recurrent posterior instability (subluxation) of the shoulder. J Bone Joint Surg Am.

[15] Huber H, Gerber C (1994). Voluntary subluxation of the shoulder in children. A long-term follow-up study of 36 shoulders. J Bone Joint Surg Br.

[16] Kuroda S, Sumiyoshi T, Moriishi J, Maruta K, Ishige N (2001). The natural course of atraumatic shoulder instability. J Shoulder Elb Surg.

[17] Moroder P, Minkus M, Bohm E, Danzinger V, Gerhardt C, Scheibel M (2017). Use of shoulder pacemaker for treatment of functional shoulder instability: proof of concept. Obere Extrem.

